# Acute organophosphorus toxicity in a regional hospital in Johannesburg, South Africa: A retrospective chart review

**DOI:** 10.1016/j.afjem.2023.04.002

**Published:** 2023-04-26

**Authors:** Vanessa Khonje, Jedd Hart, Jakus Venter, Saisha Deonarain, Saul Grossberg

**Affiliations:** a Emergency Department, Thelle Mogoerane Regional Hospital, Vosloorus, Gauteng, South Africa; b Division of Emergency Medicine, Faculty of Health Sciences, University of Witwatersrand, Gauteng, South Africa

**Keywords:** Organophosphorus toxicity, Atropinisation dose, Atropine toxicity, Emergency department, Cholinesterase levels

## Abstract

**Introduction:**

Intentional and accidental organophosphorus exposures pose a significant healthcare-related burden on South African communities. This study will review the demographics, characteristics and clinical course of patients presenting with features of acute organophosphorus toxicity to a regional Emergency Centre in Johannesburg, South Africa.

**Methods:**

This was a retrospective chart review of all patients treated for possible acute organophosphorus toxicity from January 2020 to August 2021.

**Results:**

A total of 205 patients were identified of which 134 patients were included in the study. The median age was 26 years with a male predominance (male= 56%, female=44%). 109 patients (81.3%) survived, 18 patients (13.4%) demised and the outcome of 7 patients (5.2%) was unknown. The median hospital length of stay was 8 days, (IQR= 5-13 days), and the longest hospital stay was 37 days in ICU. Atropinisation dose was significantly higher for intubated patients (median=140.0mg; IQR=90mg-219.5mg) compared to patients who were not intubated (median=60mg; IQR=20.5mg-120mg, *p* < 0.05). The length of stay was significantly higher for intubated patients (median=11 days; IQR=7-15 days) compared to patients who were not intubated (median=5 days; IQR=3-8 days, *p* < 0.00). There was a moderate positive correlation between atropinisation dose and length of stay (Correlation coefficient = 0.37, *p* < 0.00). There was a moderate negative correlation between atropinisation dose and cholinesterase level (Correlation coefficient= - 0.39, *p* < 0.00). Of those reported to have adverse effects 78.6%, were related to atropine toxicity.

**Conclusion:**

Our study shows a high mortality rate secondary to organophosphorus toxicity. Significant exposures and thus higher doses of atropine were associated with increased length of stay and need for intubation. We found a high incidence of atropine-related adverse effects. More studies are needed to further establish the balance between the therapeutic and adverse effects of high-dose atropine as a treatment modality for organophosphorus toxicity.

## African relevance

• The public health burden of organophosphate toxicity in South African communities.
• The importance of demonstrating how organophosphate toxicity is managed, the adverse effects and outcomes that need addressing.
• To emphasize the importance of pesticide regulation in the country to decrease exposure and addressing social determinants that make it use a necessity.

## Introduction

Organophosphorus toxicity represents a significant burden on the healthcare systems in developing countries. Few studies have examined the effects of intentional or accidental organophosphorus exposures in developing countries [1,2]^.^ Intentional exposure to pesticides is a common method of suicidal behaviour in developing countries and carries a mortality rate of up to 20%. Studies in Taiwan, India and China suggest pesticide ingestion, in particular exposure to organophosphorus chemicals, is the most common method of suicidal behaviour [1,3,4]. Studies done in South Africa suggest similar findings. Between 1997 and 2016, pesticide exposure was the second most common method of suicidal behaviour [5]. According to surveillance data published by the Tygerberg Poisons Information Centre in 2018, exposure to insecticides accounted for the most poisoning events in the non-drug category [6]. Veale *et al* reported similar findings, where pesticide exposures contributed to 52.7% of all poison exposures in 2013 [7]. Another study done in Tshwane; South Africa reported that intentional exposures (51.7%) outweighed accidental exposures (21.7%) [8]. Acute organophosphorus toxicity results in long, more complex hospital stay in the intensive care unit/ high-care settings with up to 71% of deaths occurring in medical centres post exposure [9], [10], [11].

A key contributing factor to the major disease burden is the ease of access to pesticides in developing countries. South Africa has an informal and unregulated street pesticide market. These hazardous pesticides are sold unlabelled and packaged in granular or liquid form. They are sold for domestic pest control by street vendors at affordable prices [12]. Unique to South Africa's landscape are townships and informal settlements with low-income housing, low household incomes and poverty [13]. Mental health conditions are also common amongst South Africans, with limited access to mental health care services in low socio-economic areas [14]. The combination of poor mental health support and easy access makes organophosphates a popular self-harm option [15].

The diagnosis of organophosphorus toxicity is a clinical diagnosis, identified by the presence of muscarinic and nicotinic symptoms [16,17] and significant toxic exposure is confirmed by a 25% decrease in serum pseudocholinesterase levels [18]. The acute management of organophosphorus toxicity is largely symptomatic, with the administration of atropine, pralidoxime or obidoxime as the main antidotes, combined with adjuncts such as glycopyrrolate, antipsychotics, benzodiazepines, and magnesium sulphate [19]. Incremental bolus administration of atropine, followed by a maintenance infusion have been shown to reduce mortality from organophosphorus exposures by up to 16% [20].

Thelle Mogoerane Regional Hospital has a high incidence of severe organophosphorus exposures, with limited resuscitation capacity. This led to a modified local rapid atropinisation protocol, with higher starting doses of atropine. Therefore, our study objectives are to describe the demographics, characteristics and clinical course of patients presenting with organophosphorus toxicity. We also aim to describe the association between mortality and various treatment aspects, including high-dose atropine. We also aim to describe the adverse effects of high-dose atropine use.

## Methods

We conducted a retrospective chart review of all patients presenting to the emergency centre at Thelle Mogoerane Regional Hospital (12390 Nguza Street, Vosloorus 1475, Gauteng, South Africa) with symptoms suggestive of cholinergic toxidrome or a history of suspected organophosphorus exposure, over a 20-month period from January 2020 to August 2021. Files were included if patients had reported symptoms of cholinergic toxidrome who received rapid atropinisation as per the local protocol, and were admitted to a high acuity care unit (Resuscitation Unit, High Care Unit, Intensive Care Unit). Files were excluded for patient ages under 12, missing patient files and multiple suspected ingestions as determined by patient, family or other witnesses’ history. Multiple suspected ingestions were excluded so as not to obscure data aimed at describing organophosphorus toxicity lastly if the patient was admitted to a standard care unit. Missing data in patient files were included in the study and were accounted for in the results. Patient files were independently reviewed by the study authors, and data was extracted using a custom-made secure data collection tool. The following data points were collected: patient demographics, presenting symptoms, dose of atropine received, intubation required, length of hospital stay, mortality, adjuncts received, atropine adverse effects, other complications related to treatment and serum cholinesterase level. A cholinesterase level of 25% below the lower limit of normal (4620-11500 U/L) was used as a diagnostic marker of significant organophosphate exposure. Our local protocol uses 10mg/ml atropine, in 10ml vials, produced by Dr Franz Kohler Chemie GmbH and imported from Germany. This formulation is authorised for use via Section 21 application by the South Africa Health Products Regulation Authority. The local protocol for rapid atropine administration is as follow: Administration of an initial 15mg atropine intravenous bolus, followed by incremental 15mg intravenous bolus administration every 3-5min until resolution of cholinergic respiratory symptoms. After rapid atropinisation, a maintenance atropine infusion, calculated at 20% of the total atropine dose is started. Oxime based therapeutic agents are unavailable in South Africa and were not used in this study. Permission to collect data was approved by the hospital CEO and Human Research Ethics Committee (Medical), the faculty of Health Sciences of the University of Witwatersrand. (M210916)

Categorical data is reported as descriptive statistics using frequencies and percentages. Parametric and non-parametric variables were determined by skewness, and are respectively described using means and standard deviations, or medians and interquartile ranges. The association between categorical variables were examined using Pearson's chi-square test. Association between continuous variables was examined using Spearman's rank correlation coefficient. A 95% confidence interval and a < 0.05 p-value was considered statistically significant. Data was analysed using IBM SPSS version 25.

## Results

Over the 20-month period, 205 patient encounters were identified as meeting the inclusion and exclusion criteria. 71 patient files were not available for data analysis, and 134 patient files were included in the data set. Table 1 contains the baseline demographic and other patient outcomes.

**Table 1:** Baseline demographics and patient outcomes.

Characteristics	*N* (%)
Sex	
Male	75 (56)
Female	59 (44)

Age	
12-20	40 (30)
21-30	53 (40)
31-40	23 (17)
41-50	6 (4)
51-60	7 (5)
61-70	3 (2)
71-80	1 (1)
91-100	1 (1)

Adjunct therapy used	
Risperidone	59 (44)
Midazolam	55 (41)
Magnesium Sulphate	31 (23.1)
Haloperidol	22 (16.4)
Glycopyrrolate	17 (12.7)
None	29 (21.6)

Cholinesterase level (U/L)	
100-500	89 (66.4)
500-1000	11 (8.2)
1000-5000	10 (7.5)
5000-10000	7 (5.2)
>10000	3 (2.2)
Not done	14 (10.5)

Intubation	
Yes	72 (53.7)
No	57 (42.5)
Unknown	5 (3.7)

Mortality	
Survival	109 (81.3)
Death	18 (13.4)
Unknown	7 (5.2)

**Fig. 1:**
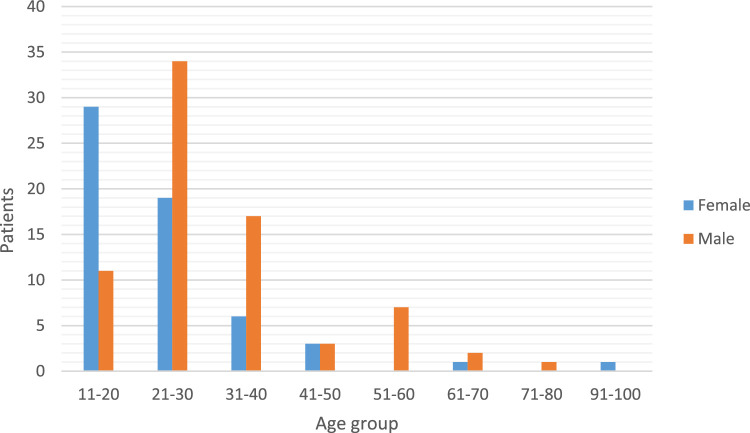
Age and sex distribution.

The median age was 26 years (IQR 19-34), the youngest patient was a 13-year-old and the oldest was a 99-year-old. 70% of organophosphorus exposures occurred in the age group 12–30 years of age. A male predominance was noted with 75 males (56%) and 59 females (44%) and this was true for most age categories above 21 years of age. In the age group 12-20, females (73%) were more common (Fig. 1).

**Fig. 2:**
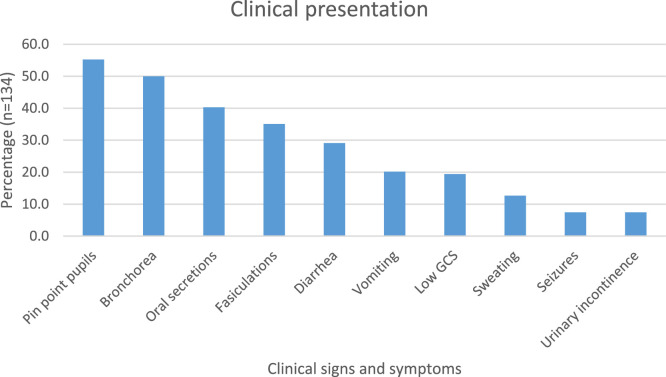
Prevalence of cholinergic and nicotinic symptoms.

The median length of stay was 8 nights (IQR 5-13 days) and the longest hospital stay was 37 days. The mortality rate was 13.4%, 81.3% patients survived, while the outcome was not recorded in 5.2% of cases. The median atropinisation dose was 100mg (IQR 45-167). The median cholinesterase level (U/l) was 208.5 (IQR 129.75-545.5).The most common symptoms of cholinergic and nicotinic toxidrome recorded were pinpoint pupils (55.2%), bronchorrhea (50%), oral secretions (40.3%) and fasciculations (35.1%) (Fig. 2).

Seventy-two patients required intubation (53.7%) The indication for intubation was not reliably documented. The length of stay was significantly higher for intubated patients (Median=11 days; IQR= 7-15 days) compared to patients who were not intubated (Median=5 days; IQR=3-8 days, *p* < 0.05). The difference in mortality rate amongst intubated patients (18.2%) compared to non-intubated patients (7.1%) was not statistically significant (p=0.07). Furthermore the possible cause of death was not documented in the non-intubated group. Of the patients admitted to high acuity care units, 46 had documented complications including sepsis, recrudescence, and cardiac arrest. The rate of complications was not reliably recorded, and thus was not included in statistical analysis.

One hundred and twenty-five patients had a documented atropinisation dosage, while nine patients had no documented doses. There was a moderate positive correlation between atropinisation dose and length of stay (Correlation coefficient = 0.37, p=0.00). A moderate negative correlation was noted between atropinisation dose and cholinesterase levels (Correlation coefficient= - 0.39, p=0.00). The atropinisation dose was significantly higher for intubated patients (Median=140.0mg; IQR=90-219,5mg) compared to patients who were not intubated (Median=60mg; IQR=20.5-120mg) (*p* < 0.05).

One hundred and five patients (78%) received adjuvant treatment, the most commonly used were risperidone (44%), midazolam (41%) and magnesium sulphate (23.1%). Only 70 patients had documented treatment complications, of which 55 patients (78.6%) were documented adverse effects from atropine toxicity. The most common symptoms of atropine toxicity were confusion (70%), tachycardia (13.4%) hallucinations (5.7%), hyperthermia (4.3%) and seizures (2%).

120 patients had cholinesterase levels (89%) and 14 (10.44%) had no cholinesterase levels done. The cholinesterase level was significantly different between survivors (Median=100.0U/L; IQR=138-634U/L) and non-survivors (Median=125.5U/L; IQR= 100-204 U/L, p=0.01).

## Discussion

Our data shows an increased burden of acute organophosphorus toxicity at our facility. 205 patient encounters in 20 months are markedly higher than other hospitals from previous studies done in the region. A retrospective study done at Chris Hani Baragwanath hospital saw 129 patients admitted to high acuity wards over 2012-2015 [11] and 207 cases (including children) were reported to the Tshwane District Surveillance office from 2012-2014 [8].

Our study looked at adolescent and adult presentations of acute organophosphorus toxicity, 70% were below 30 years comparable to a study done in Pakistan where 80% were below 30 years [21]. Our study did not examine the triggers for ingestion or whether they were intentional or accidental exposures, though most data demonstrate more intentional exposures than not [9,22]. During our study period, samples were collected at street vendors and shopping centres in the area. Ethion and Terbufos, both organophosphorus chemicals, were detected [23]. The WHO classify both these chemical compounds as class 2 (moderately hazardous) and class 1a (extremely hazardous) respectively [24]. Within South Africa commonly ingested compounds include: chlorpyriphos, terbufos and methamidophos [7,9,12]. In Asia compounds include: Chlorpyrifos, Dichlorvos and parathion [25,26,27] classified as either class 2, 1b (highly hazardous) and class 1a in order. Exposure to such hazardous compounds contribute to the severe clinical presentations seen.

Muscarinic symptoms were most common, and the most common documented nicotinic sign was fasciculations (Fig. 2), comparable to studies done in South Africa, Bangladesh and Pakistan [11,20,28]. This demonstrates the ease of detecting muscarinic signs versus relatively subtle nicotinic signs. Our atropine median dose was comparable to a randomised-control trial on atropine dosing in Bangladesh where the mean atropine bolus dose in each study group was 109mg and 136mg [20] (Appendix B).

Pseudocholinesterase levels were collected in all but 14 patients. The levels were collected at the time of presentation in all but 4 patients whose levels were done 2 or 4 days after presentation, each of which still had lower than 25% of the normal cholinesterase range. The cholinesterase level was significantly higher for patients who demised compared to patients who survived, with levels below 25% of the normal range in each group. This is supported by data that suggests a single cholinesterase level cannot determine patient outcome and a trend in cholinesterase levels may be more helpful to assist in prognostication [29]. Pseudocholinesterase levels are used to determine exposure to organophosphates whereas red cell cholinesterase is a good marker of poison severity [17]. In our setting pseudocholinesterase is the investigation of choice, due to difficulty in obtaining a red cell cholinesterase level through the local laboratory. Pseudocholinesterase levels can be affected by both intrinsic and extrinsic factors, which can lead to inaccurate results. Factors such as: transit time to lab, malnutrition, liver dysfunction, iron deficiency, and drug administration like morphine, succinylcholine and other induction drugs all have an influence on the measured serum pseudocholinesterase level [29]. Although these factors were not scrutinized in this study, it may have influenced the cholinesterase levels. Despite discordant data regarding the interpretation of these levels, studies have shown there is no significant association with mortality [30] and that higher levels above 1000 IU/L had significantly lower need for ventilation, vasopressor use and length of stay in a prospective study (n=37) comparing the clinical outcomes in two groups with pseudocholinesterase levels above and below 1000 IU/L [31].

Atropine adverse reactions can be difficult to identify and is done so based on exclusion, noting the effects are non-specific and may be a part of other complications in the emergency centre and high acuity wards. Only 70 files had documented side effects. Those most related to atropine toxicity found in 55 files were: confusion, hallucinations, pyrexia and tachycardia. A study looking at atropine toxicity in Sri Lanka found the most common adverse effects were hallucinations and delirium, both of which were associated with higher atropine doses of 15mg versus 3,9mg [25]. Another study in Bangladesh documented atropine toxicity to include confusion, pyrexia and absent bowel sounds with urinary retention [20]. Other symptoms that may support the diagnosis of atropine toxicity are dilated pupils, dry mucosal membranes, flushed skin and dermatitis [32]. Risperidone was the most commonly used adjuvant therapy. It was used for patients with agitation, confusion and psychotic symptoms such as: hallucinations. Agitated delirium has been well described in the literature occurring as a result of many factors: the organophosphorus chemical itself, atropine toxicity and hypoxia [17,19]. Thus the standard care protocol recommends benzodiazepines [17,19], for which midazolam was most commonly used. However neuro-behavioural changes can occur post-acute and chronic exposure to organophosphorus chemicals: which may present as anxiety, psychosis, depression, extra-pyramidal side effects and impaired memory [33,34,35]. Early case reports described the short-term use of chlorpromazine [36] with more recent case reports using quetiapine and olanzapine in managing the neuro-behavioural effects of organophosphorus exposure [33,37].

Acute organophosphate toxicity contributes to a greater high-care and ICU burden through increased length of stay, ICU complications and cost [10,38]. The atropinisation dose and length of stay was significantly higher for intubated patients (53.7%), and the mortality increased further for intubated patients suggesting poorer outcomes in this group.

## Limitations

As a retrospective chart review, our study had major limitations. 71 patient files were not found and needed to be excluded from the study sample. Many of the patient records that were included, had missing data. To mitigate this limitation in future, a prospective study design should be used in order to determine the association between mortality, severity of organophosphorus toxicity presentations, its treatment and complications. We report only on data collected at a single, well resourced, regional hospital. Our results may not be applicable to under resourced, district level or community health facilities. Recall bias may have been possible through the interpretation of events that occurred after each patient presentation and their outcome. Reporting bias is also likely, especially with adverse effects, as staff may fear punitive action if adverse events are reported. The data was also collected during the COVID19 pandemic and therefore the number of presentations may not have been reflective of the community patient load due to COVID19 movement restrictions.

## Conclusion

Organophosphorus exposures represent a significant health care burden on health care systems in developing countries, and our facility is not different. Our study highlights the high mortality rate from organophosphorus exposure. Many of our patients required intubation and prolonged hospital stay, increasing the risk of morbidities. Our data also support the therapeutic effects of rapid atropinisation, and at the same time highlights the potential adverse effects from using high doses of atropine. As practitioners caring for patients with acute organophosphate toxicity, maintaining a balance between the therapeutic and adverse effects of atropine is important to patient safety. More studies are needed to further establish treatment and safety protocols for the administration of high dose atropine in organophosphorus toxicity.

## Recommendations

Moving forward, our research can be improved with the following:

1. Develop an acute organophosphorus toxicity data collecting tool for a future prospective study.
2. Improve surveillance through notification of disease.
3. Further investigation of atropine dose regimes, without being given oximes.

## Dissemination of results

The results were shared with the Emergency Medicine staff through a PowerPoint presentation.

## Authors’ contributions

Authors contributed as follow to the conception or design of the work; the acquisition, analysis, or interpretation of data for the work; and drafting the work or revising it critically for important intellectual content: VK contributed 50%, JV and JH contributed 20%, SD and SG contributed 10%. All authors approved the version to be published and agreed to be accountable for all aspects of the work.

VK - Conceptualization, Data curation, Funding acquisition (self-funded), Project administration, Writing - original draft, Writing - review and editing. JV - Methodology, Writing - review and editing, visualisation, Formal analysis JH - Supervision, Validation, Writing - review and editing, Resources and visualisation. SD and SG - Formal analysis, Software, Data curation.

## Declaration of Competing Interest

The authors in this study declare no conflict of interest. Therefore there was no associations with commercial entities that worked with or had interest in the submission of the manuscript. There were no financial or non-financial associations to the submitted manuscript.
