# Supplementary material for: Acute organophosphorus toxicity in a regional hospital in Johannesburg, South Africa: A retrospective chart review
Source: Afr J Emerg Med. 2023 Apr 26;13(2):104–8. doi: 10.1016/j.afjem.2023.04.002 (PMC10160343; doi:10.1016/j.afjem.2023.04.002)
Supplement: Supplementary file 1 [file mmc1.docx]

# **Appendix B**

**Table 3** Comparison of organophosphorus toxicity data

| **Country** | **Study** | **Total number of patients** | **Sex (M/F) %** | **Atropinisation, median (mg)** | **Oximes given** | **Atropine toxicity (%)** | **Intubation %** | **Length of stay (days)** | **Cholinesterase levels median (IU/L)** | **Outcome**  **(demised) %** |
| --- | --- | --- | --- | --- | --- | --- | --- | --- | --- | --- |
| **South India**^[27]^ | (Reddy et al., 2020) | 441 | 69.1/30.8 | 454 | Yes | - | 41 | 11 | 601.5 | 14.18 |
| **India**^[31]^ | (Gagarin and Rajagopal, 2020) | 100 | 80/20 | - | - | - | 40 | - | <1000: 6^¥^  1001-2000: 18  2001-5000: 68  >5000: 8 | 25 |
| **Korea**^[26]^ | (Kang et al., 2009) | 68 | 56/44 | - | Yes | - | 51 | - | 209 | 19 |
| **China**^[34]^ | (Tang et al., 2016) | 71 | 61.97/32.39 | - | No | - | - | Survived: 12.7  Demised: 6,7* | Survived:1205.6  Demised: 556.7* | 16.9 |
| **South Africa**^[11]^ | (Bruins, Menezes and Wong, 2019) | 129 | 68.2/31.8 | - | No | - | 99^◦^ | 3 | 200 | 5.4 |
| **South Africa**^[10]^ | (Omar et al. 2021) | 41 | 54/46 | - | No | - | 95^◦^ | 5 | - | - |
| **Pakistan**^[28]^ | (Ather et al. 2008) | 2708 | 51/48 | - | Yes | - | 4 | - | <4500IU: 1527^¥^  4500-10000: 1604  >10000: 303 | 5 |
| **Bangladesh**^[20]^ | (Abedin et al. 2012) | 156 | 60/40 | Group A: 109*  Group B:136* | Yes | 20 | - | - | - | 15.5 |
| **Taiwan**^[22]^ | (Tsai et al. 2007) | 75 | 66/34 | 27* | Yes | - | 28 | Mild cases: 5.9  Severe cases: 11.9  Life threatening: 13.9 | Mild cases: 2034  Severe cases: 557.5  Life-threatening cases: 674.5 | 8 |
| **Hong Kong**^[30]^ | (Yun et al. 2012) | 55 | 62/38 | - | - | - | Survived: 81  Deceased: 100 | Survived: 20  Deceased: 8 | Survived: 4338.6  Deceased: 2729.37 | 14.5 |
| **North India**^[29]^ | (Chaudery et al. 2013) | 70 | 70/30 | Group A: 509.17  Group B: 566 | Yes in group B only | - | Group A: 48.5  Group B: 60 | Group A: 7.05  Group B: 7.65 | 3154.16 U/L* | 11 |
| **Sri Lanka**^[25]^ | (Perera et al. 2008) | 272 | 64/36 | Ad hoc: 15  Titrated regimen: 3.9 | Yes | 20 | Ad hoc: 1  Titrated doses: 6 | - | - | 9 |
| *Mean was used instead of median^, ¥^Actual number^, ◦^ICU/HC data only, - Not documented | | | | | | | | | | |
